# SATB1 is overexpressed in metastatic prostate cancer and promotes prostate cancer cell growth and invasion

**DOI:** 10.1186/1479-5876-11-111

**Published:** 2013-05-04

**Authors:** Lijun Mao, Chunhua Yang, Junqi Wang, Wang Li, Rumin Wen, Jiacun Chen, Junnian Zheng

**Affiliations:** 1 Department of Urology, Affiliated Hospital of Xuzhou Medical College, Xuzhou, 221002, China; 2 Jiangsu Key Laboratory of Biological Cancer Therapy, Xuzhou Medical College, Xuzhou, 221002, China

**Keywords:** SATB1, Prostate cancer, Metastasis, Invasion, Proliferation

## Abstract

**Background:**

Special AT-rich sequence binding protein 1 (SATB1) is a nuclear factor that functions as the global chromatin organizer to regulate chromatin structure and gene expression gene expression. SATB1 has been shown to be abnormally expressed in various types of cancer. However, the expression and role of SATB1 in prostate cancer remain unclear.

**Methods:**

120 cases of prostatic carcinoma and 60 cases of benign prostate hyperplasia were analyzed for SATB1 expression by immunohistochemistry. LNCaP, DU-145, and PC3 prostate cancer cells were examined for SATB1 expression by Western blot analysis. Cell proliferation and invasion was evaluated by CCK8 and transwell invasion assay, respectively.

**Results:**

SATB1 staining was stronger in prostatic carcinomas with metastasis than in those without metastasis, but was absent in benign prostate hyperplasia. Furthermore, SATB1 expression was positively correlated with bone metastasis and the Gleason score. SATB1 overexpression promoted the proliferation and invasion of LNCaP cells while SATB1 knockdown inhibited the proliferation and invasion of DU-145 cells.

**Conclusions:**

These findings provide novel insight into oncogenic role of SATB1 in prostate cancer, suggesting that SATB1 is a promising biomarker and therapeutic target for prostate cancer.

## Background

Prostate cancer is the most frequent cancer among men over 50 years old in industrialized countries. With the development of PSA screening, MRI imaging and new prostate biopsies protocols, the accuracy of detection and localization of prostate tumors has been increased, but still 5% of cases present with metastatic lesions at the time of diagnosis [1]. The most common site of metastasis for prostate cancer is bone, and frequently metastasis is symptomatic, with pain, debility, and functional impairment [2]. Therefore, it is important to investigate the molecular mechanisms underlying the progression and metastasis of prostate cancer to provide better strategies for the prevention and therapy of prostate cancer.

Overexpression of embryonic transcription factors has been linked to cancer development and progression. Inappropriate expression of these transcription factors is thought to reinstitute developmental programs out of context, and contributing to tumor formation and progression. Special AT-rich sequence binding protein 1 (SATB1) is a nuclear factor that functions as the global chromatin organizer to regulate chromatin structure and gene expression [3]. SATB1 has been shown to be abnormally expressed in various types of cancer and is proposed as an oncogene which promotes malignancy [4-6]. However, the expression and role of SATB1 in prostate cancer remain unclear.

In this study, we performed immunohistochemistry analysis to examine the expression of SATB1 in clinical prostatic carcinoma samples and analyze the correlation of SATB1 expression with the clinicopathological features of prostate cancer. Furthermore, we used a panel of prostate cancer cell lines including LNCaP, DU145, and PC3 to investigate the function of SATB1 in prostate cancer cell proliferation and invasion.

## Methods

### Tissue microarray and immunohistochemistry

Total 120 cases of prostate carcinoma were enrolled in the present study. The patients had undergone surgical resection at the affiliated hospital of Xuzhou Medical College between 2006 and 2012. Among the 120 cases of prostate carcinoma, 60 cases had metastasis to the bone including one case with additional metastasis to the lung and two cases with additional metastasis to lymph node. For the other 60 cases without metastases, they were newly diagnosed and had no recurrence or bone disease. 60 cases of benign prostate hyperplasia were also included in this study, including 20 from open prostatectomies and 40 from transurethral plasmakinetic enucleation of prostate. The immunohistochemical analysis was performed by pathologists at the affiliated hospital of Xuzhou Medical College to detect SATB-1 expression in 60 cases of prostatic carcinoma with metastases, 60 cases of prostatic carcinoma without metastases, and 60 cases of benign prostate hyperplasia. Briefly, the samples were fixed in 40 g/L paraformaldehyde, wax-embedded and cut into 4 μm serial sections. Next, endogenous peroxidases were quenched and the sections were washed carefully with 0.01 M phosphate buffered saline (PBS) for three times. The sections were blocked with 2% goat serum in 0.01 M PBS at room temperature for 1 h, then incubated with rabbit polyclonal anti-SATB1 antibody (1:500 dilution, NB110-17780, Novus Biologicals, Littleton, CO, USA) overnight at 4°C. Afterwards, the sections were incubated with goat anti-rabbit horseradish peroxidase-conjugated secondary antibody and avidin-biotin complex followed by diaminobenzidine (Vector ABC, Burlingame, CA, USA). For negative control, tissue was incubated with rabbit IgG serum.

The immunohistochemical staining was scored by pathologists based on the intensity and percentage of cells with SATB1 nuclear staining as follows: score 0, negative nuclear staining of almost all tumor cells; score 1, weak nuclear staining of almost all tumor cells; score 2, moderate nuclear staining of more than 50% tumor cells or strong nuclear staining of more than 5% tumor cells. Samples that could not be interpreted or missed most of the tumor tissue were given a score of not applicable (N/A). Scoring of the tissue microarray was performed by three independent observers. Significance of correlation between SATB1 staining and histopathological factors was determined using Pearson’s chi-squared (*x*2) test.

### Cell culture and transfection

The human prostate cancer cell line LNCaP, DU-145, and PC-3 were obtained from Institute of Biochemistry and Cell Biology, Chinese Academy of Sciences (Shanghai, China) and cultured in RPMI-1640 medium (Gibco, USA) supplemented with 10% fetal calf serum (Hyclone, USA), penicillin and streptomycin at 37°C in 5% CO_2_. Cells were regularly passaged to maintain exponential growth. SATB1 expression vector pcDNA3.1-SATB1 or SATB1 specific shRNA pSilencer3.1-SATB1 (the sequences of the oligos that targeted SATB1 were as follows: 5′-CACCGGATTTGGAAGAGAGTGTCTTCAAGAGAGACACTCTCTTCCAAATCCTTTTTTG-3′, 63 bp) was constructed and transfected into cells using lipofectamin 2000 (Invitrogen, USA) according to the manufacturer’s instructions. Cells transfected with pcDNA3.1 or pSliencer3.1 empty vector were used as negative control. The cells were harvested at different time points after transfection for the following experiments.

### Western blotting

Cells were collected and lysed with RIPA lysis buffer (Santa Cruz Biotechnology, USA). The cell lysates were collected after centrifugation and the protein concentration was quantified by bovine serum albumin (BSA) method. Equal amount of protein was loaded and separated on 10% SDS-PAGE, and then transferred to nitrocellulose membranes (Millipore, USA). The membranes were blocked in 3% BSA for 2 h and then incubated with SATB1, SATB2 or β-actin antibody (Santa Cruz Biotechnology, USA) at 4°C overnight. After three times of washing with TBST,the membranes were incubated with HRP-conjugated secondary antibodies (Santa Cruz Biotechnology, USA) for 1 h at room temperature. The membranes were developed using ECL kit (Santa Cruz Biotechnology, USA) and exposed to X-ray film. β-actin was used as loading control. The density of the bands on the membrane were scanned and analyzed with an image analyzer (LabWorks Software, UVP Upland, CA, USA).

### Cell proliferation assay

The proliferation of DU145 cells was examined using CCK-8 kit (Tongren Shanghai Co. China) according to the manufacturer’s instructions. Briefly, the cells were seeded into 96-well plates with 5 × 10^3^ cells/well and cultured for 48 h. Then 10 μl CCK-8 solution was added to each well and incubated for 1 h. The absorbance (A) at 450 nm was measured using a microplate reader. Results were representative of three individual experiments in triplicate.

### In vitro invasion assay

The invasion ability of DU145 cells was detected using 24-well transwell cell culture chambers (6.5 mm diameter, 8.0 μm pore size, polycarbonate membrane, Corning, USA). Briefly, an aliquot of 10^5^ cells were added into the upper chamber with 100 uL serum-free medium. The lower chamber was filled with 0.6 ml medium containing 10% FBS. After 20 h incubation at 37°C in 5% CO2 incubator, the cells on the upper surface of the filters were removed by wiping with a cotton swab. The filters were fixed in 4% paraformaldehyde and stained with Crystal Violet. The stained cells were counted under a microscope in five randomly selected fields. At least three chambers from three different experiments were analyzed.

### Statistical analysis

The results were expressed as mean ± SD. Statistical analysis was performed using Student’s *t* test or a 1-way or 2-way analysis of variance (ANOVA) test followed by Tukey’s test. P < 0.05 was considered to be statistically significant.

## Results

### SATB1 expression is correlated with clinicopathological features of prostate cancer

**Figure 1:**
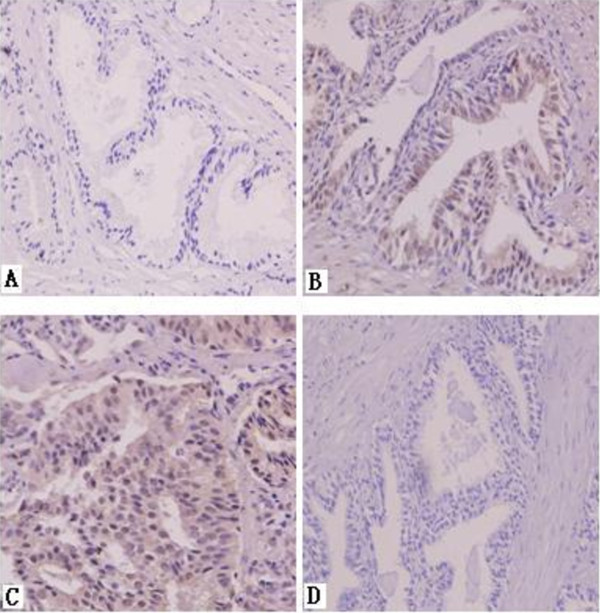
**SATB1 immunohistochemistry staining. A**, benign prostate hyperplasia. **B**, prostatic carcinoma with metastasis. **C**, prostatic carcinoma without metastasis. **D**, negative control. Magnification: 400 × .

SATB1 expression in prostate cancer tissues was examined by immunohistochemistry. The results showed that the positive rate of SATB-1 staining was 86.7% (104/120) in 120 cases of prostatic carcinoma and 0% (0/60) in 60 cases of benign prostate hyperplasia. SATB1 was not stained in cells of benign prostate hyperplasia (Figure 1A), but was positively stained in the nucleus of prostate cancer cells (Figure 1B,C). In addition, SATB1 staining was stronger in cells of prostatic carcinoma with metastasis than in those of prostatic carcinoma without metastasis.

Furthermore, we analyzed the correlation of SATB1 expression with clinicopathological features of prostate cancer. The results shown in Table 1 demonstrated that SATB1 expression was positively correlated with the bone metastasis and the Gleason score (P < 0.05), but not with patient age or the concentration of prostate-specific antigen in blood serum (P > 0.05). These data suggest that SATB-1 expression is closely related with the occurrence and development of prostate cancer.

**Table 1:** SATB1 expression and clinicopathologic parameters of prostate cancer

**parameter**	**cases**	**SATB1 expression**				**P value**
		-	+	++	+++	
metastases						
+	30	2	4	14	10	
-	30	6	9	12	3	0.006 <0.05
Age (year)						
≤70	37	5	8	16	8	
>70	23	3	5	10	5	0.837 >0.05
PSA (ng/ml)						
≤20	21	4	3	8	6	
>20	39	4	10	18	7	0.713 >0.05
Gleason score						
2-4	17	4	7	5	1	
5-7	20	3	2	11	4	
8-10	23	1	4	10	8	0.009 <0.05

### SATB1 expression level is correlated with the invasion ability of prostate cancer cells

**Figure 2:**
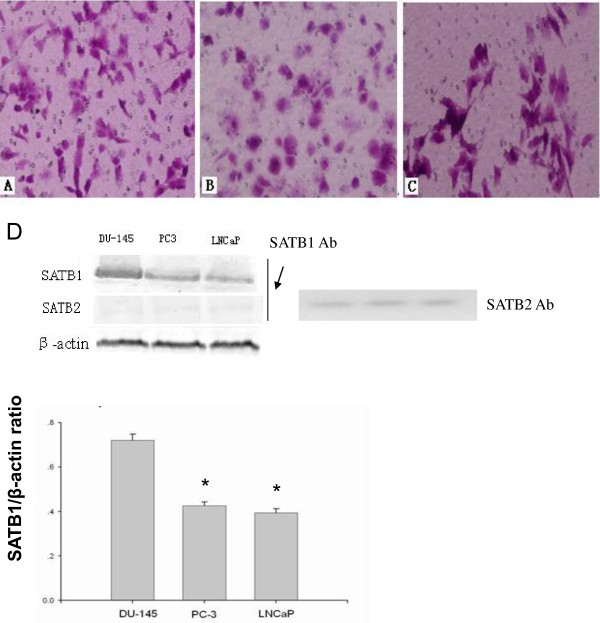
**SATB1 expression is correlated with the invasion ability of prostate cancer cells. A**-**C**: The invasion of LNCaP, DU-145 and PC-3 cells was measured by Transwell chamber. **A**, DU-145. **B**, PC-3.**C**, LNCaP. Magnification: 200×. **D**: Western blot analysis of the relative protein level of SATB1 in LNCaP, DU-145 and PC-3 cells (n = 3). β-actin served as loading control. *P < 0.05 compared to DU-145 cells. Note that SATB1 antibody could not detect SATB2 protein in the cell lysates.

Since SATB1 expression is positively correlated with the bone metastasis of prostate cancer, we hypothesized that SATB1 expression may confer high invasion ability in prostate cancer cells. Therefore, we selected several prostate cancer cell lines LNCaP, DU-145 and PC-3 with different invasion ability. Transwell invasion assay demonstrated that the number of cells that invaded the membrane was 98.6 ± 8.5 in DU-145 cells, 68.5 ± 7.1 in PC-3 cells, and 42.7 ± 4.5 in LNCaP cells (Figure 2A-C). Western blot analysis showed that the ratio of SATB1/β-actin was 0.72 ± 0.03, 0.42 ± 0.02, 0.39 ± 0.02 in DU-145, PC-3, and LNCaP cells, respectively (Figure 2D). These results demonstrate that the invasion ability of these three cancer cell lines was consistent with the protein expression level of SATB1, indicating the potential role of SATB1 in prostate cancer metastasis.

### Silencing of SATB1 inhibits the invasion and growth of DU-145 cells

**Figure 3:**
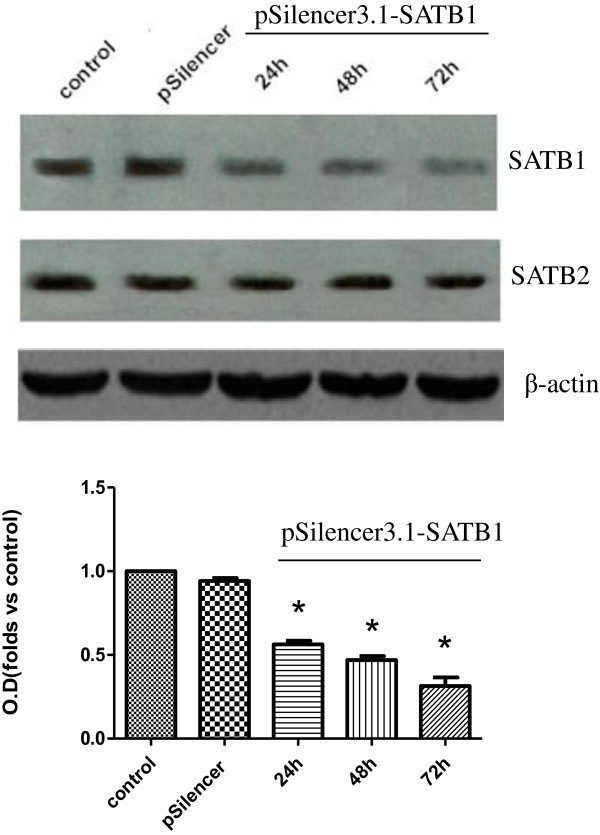
**Silencing of SATB1 in DU-145 cells.** DU-145 cells were untransfected (control), transfected with pSliencer3.1 control vector or transfected with pSilencer3.1-SATB1 vector for 24 h, 48 h, or 72 h. The protein levels of SATB1 and SATB2 were detected by Western blot analysis (n = 3). β-actin served as loading control. *P < 0.05 compared to cells transfected with pSliencer3.1.

To investigate the role of SATB1 in the invasion of prostate cancer cells, we employed the loss of function approach to knockdown SATB1 expression in DU-145 cells in which SATB1 expression is relatively high. Western blot analysis showed that after the transfection of pSilencer3.1-SATB1 into DU145 cells, protein level of SATB1 decreased in a time dependent manner and reached 31.4 ± 9.0% of the control at 72 h after transfection, while protein level of SATB2 remained unchanged (Figure 3). These results demonstrate that SATB1 shRNA construct could knockdown SATB1 expression in DU145 cells effectively and specifically.

**Figure 4:**
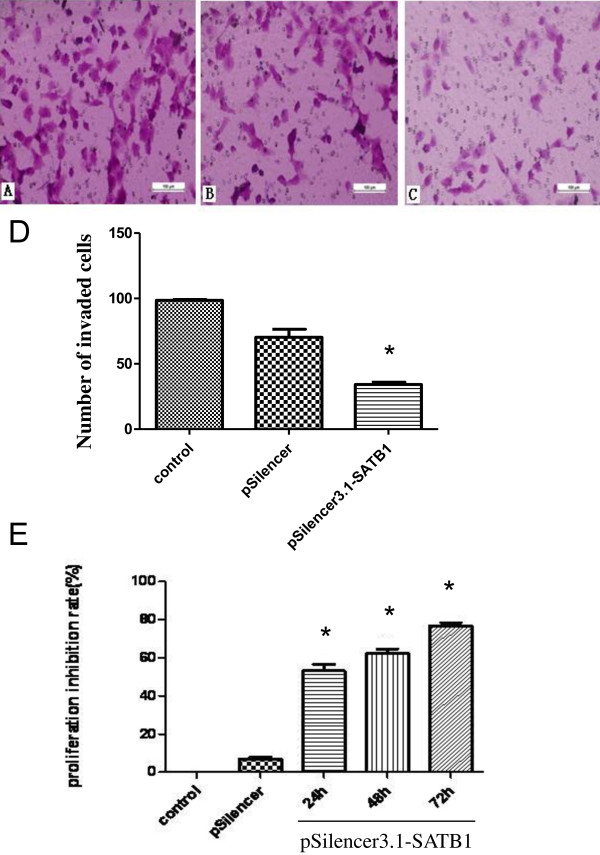
**Silencing of SATB1 inhibits the invasion and growth of DU-145 cells. A**-**D**: The invasion of DU-145 cells was measured by Transwell chamber. A, DU-145 cells untransfected. **B**, DU-145 cells transfected with pSliencer3.1. **C**, DU-145 cells transfected with pSilencer3.1-SATB1. Magnification: 200×. **D**: Quantitative analysis of the number of invaded cells as shown in **A**-**C** (n = 3). **E**: The proliferation of DU-145 cells was determined by CCK8 assay (n = 3). The cell proliferation inhibition rate was calculated for DU-145 cells at 24 h, 48 h and 72 h after transfection with pSilencer3.1-SATB1. *P < 0.05 compared to cells transfected with pSliencer3.1.

Next we performed transwell invasion assay and the results demonstrated that the number of cells that invaded the membrane was 96.5 ± 6.5 in untransfected DU-145 cells, 90.0 ± 5.4 in DU-145 cells transfected with control vector, and 34.2 ± 4.2 in DU-145 cells transfected with SATB1 shRNA (Figure 4A-D).

In addition, we performed cell proliferation assay using CCK-8 kit and the results showed that the proliferation of DU-145 cells was inhibited at 53.2 ± 5.8%, 62.3 ± 4.0%, and 76.7 ± 3.0% at 24 h, 48 h, 72 h after transfecion of SATB1 shRNA, respectively (Figure 4E). Collectively, these results suggest that silencing of SATB1 inhibits the invasion and growth of prostate cancer cells.

### Overexpression of SATB1 promotes the invasion and growth of LNCaP cells

**Figure 5:**
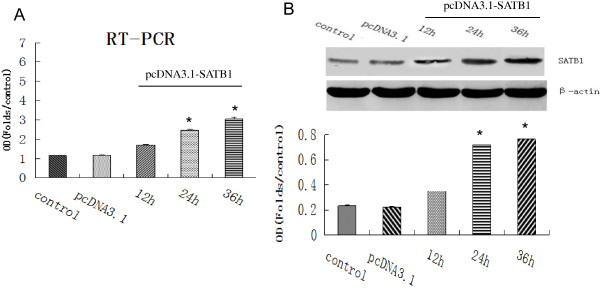
**Reconstitution of SATB1 expression in LNCaP cells.** LNCaP cells were untransfected (control), transfected with pcDNA3.1 control vector or transfected with pcDNA3.1-SATB1 vector for 12 h, 24 h, or 36 h. **A**. The mRNA level of SATB1 was detected by RT-PCR (n = 3). **B**. The protein level of SATB1 was detected by Western blot analysis (n = 3). β-actin served as loading control. *P < 0.05 compared to cells transfected with pcDNA3.1.

To further confirm the role of SATB1 in the invasion of prostate cancer cells, we employed the gain of function approach to overexpress SATB1 in LNCaP cells in which SATB1 expression is relatively low. RT-PCR analysis showed that after the transfection of pcDNA3.1-SATB1 into LNCaP cells, mRNA level of SATB1 increased in a time dependent manner and reached 304.54 ± 9.89% of the control at 36 h after transfection (Figure 5A). Similarly, Western blot analysis showed that after the transfection of pcDNA3.1-SATB1 into LNCaP cells, protein level of SATB1 increased in a time dependent manner and reached 334.74 ± 4.07% of the control at 36 h after transfection (Figure 5B). These results demonstrate that the SATB1 expression construct we designed could effectively increase SATB1 expression to a high level in LNCaP cells.

**Figure 6:**
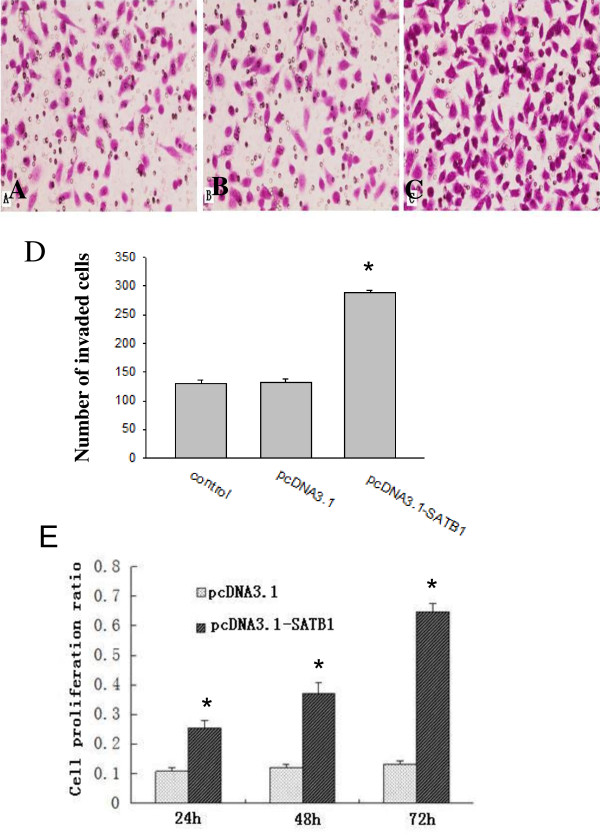
**Overexpression of SATB1 promotes the invasion and growth of LNCaP cells. A**-**D**: The invasion of LNCaP cells was measured by Transwell chamber. **A**, LNCaP cells untransfected. **B**, LNCaP cells transfected with pcDNA3.1. **C**, LNCaP cells transfected with pcDNA3.1-SATB1. Magnification: 200×. **D**: Quantitative analysis of the number of invaded cells as shown in **A**-**C** (n = 3). *P < 0.05 compared to cells transfected with pcDNA3.1. **E**: The proliferation of LNCaP cells was determined by CCK8 assay (n = 3). The cell proliferation ratio was calculated for LNCaP cells at 24 h, 48 h and 72 h after transfection of pcDNA3.1 or pcDNA3.1-SATB1. *P < 0.05 compared to corresponding cells transfected with pcDNA3.1.

Transwell invasion assay demonstrated that the number of cells that invaded the membrane was 131.0 ± 7.9 in untransfected LNCaP cells, 130.3 ± 5.5 in LNCaP cells transfected with control pcDNA3.1 vector, and 288.3 ± 4.5 in LNCaP cells transfected with pcDNA3.1-SATB1 vector (Figure 6A-D).

Furthermore, CCK-8 cell proliferation assay showed that the proliferation ratio was 25.3 ± 2.6%, 37.1 ± 3.7%, and 64.6 ± 2.9% in LNCaP cells at 24 h, 48 h and 72 h after transfection with pcDNA3.1-SATB1 vector, respectively, but was only 10.7 ± 1.2%, 12.1 ± 1.2%, and 13.3 ± 1.1% in LNCaP cells transfected with pcDNA3.1 control vector, respectively (Figure 6E). Taken together, these results suggest that SATB1 promotes the invasion and growth of prostate cancer cells.

## Discussion

As the second cause for cancer-related death, prostate cancer is a global public health problem. Unfortunately, up to now very limited information is available to discern which cases of prostate cancer are likely to remain latent, versus those that are likely to metastasize and warrant more aggressive management [7]. Therefore, there is urgent need to identify novel biomarkers of prostate cancer that could distinguish benign versus malignant or even metastatic prostate cancer.

SATB1 is an important transcription factor that plays a critical role in the early embryonic development and the formation of tissues and organs [8]. Recent studies suggest that SATB1 modulates cell proliferation and lineage development and is implicated in breast cancer [9]. However, the functional role of SATB1 in prostate cancer progression and metastasis remains elusive.

In this study we first examined the expression of SATB1 in clinical prostate cancer tissues. The immunohistochemistry analysis showed that SATB1 staining was stronger in prostatic carcinoma with metastasis than in prostatic carcinoma without metastasis, but was absent in benign benign prostate hyperplasia. Furthermore, our analysis showed that SATB1 expression was positively correlated with the bone metastasis and the Gleason score. These data suggest that SATB1 expression is crucially implicated in the progression and metastasis of prostate cancer.

To provide further evidence that SATB1 contributes to prostate cancer development, we employed several prostate cancer cell lines LNCaP, DU-145 and PC-3, and performed gain and loss of function experiments. We found that SATB1 protein level was the highest in DU-145 cells, which corresponds well with its high invasion ability. Meanwhile SATB1 protein level was the lowest in LNCaP cells, which corresponds well with its low invasion ability. Based on these results, we speculated that SATB1 expression is correlated with the invasion ability of prostate cancer cells. Next we knockdown SATB1 expression in DU-145 cells and found that the depletion of SATB1 led to decreased cell invasion and proliferation. Moreover, when we overexpressed SATB1 in LNCaP cells, we found that this resulted in increased cell invasion and proliferation. Therefore, both gain and loss of function approaches demonstrate that SATB1 promotes prostate cancer cell invasion and proliferation in vitro, and these results are consistent with the data on the clinical prostate cancer samples.

However, further studies are important to investigate the molecular mechanisms by which SATB1 promotes the malignant behaviors of prostate cancer cells. SATB1 constitutes a functional nuclear architecture that has a ‘cage-like’ protein distribution surrounding the heterochromatin. This architecture is proposed as “SATB1 regulatory network” by which SATB1 regulates gene expression via the recruitment of chromatin remodelling/modifying enzymes and transcription factors to genomic DNA [10-12]. Upon the activation of T-helper 2 cells, SATB1 becomes expressed and folds the cytokine-gene locus into dense loops for rapid induction of multiple cytokine genes [13]. In breast cancer cells, SATB1 coordinates the expression of a large number of genes to induce metastasis. Removal of SATB1 from aggressive breast cancer cells not only reversed metastatic phenotypes but also inhibited tumor growth, indicating its key role in breast cancer progression [9].,Barboro et al. reported that the interaction of SATB1 with specialized DNA sequences called matrix attachment regions (MARs) was important for the aggressive potential of prostate cancer cells [14]. In addition, a very recent study showed that knockdown of SATB1 in highly aggressive prostate cancer PC-3 M cells inhibited tumor growth and invasion along with increased E-cadherin expression, suggesting that SATB1 promotes prostate cancer aggressiveness through epithelial-mesenchymal transition [15]. Thus we speculate that SATB1 modulates gene expression to regulate the proliferation and invasion of prostate cancer cells. In this context, it is important to identify the downstream target genes of SATB1 that are involved in cell proliferation and invasion. For example, Bcl-2 and c-myc have been reported recently as the targets of SATB1 [16,17]. In our future investigations, we will examine the expression of these important SATB1 targets in prostate cancer cells, to provide novel insights into the potential oncogenic role of SATB1 in prostate cancer.

On the other hand, a recent study reported that miR-448 could target SATB1 and suppress its expression in breast cancer cells, and miR-448 was downregulated during epithelial–mesenchymal transition, an important process of tumor metastasis [18]. Therefore, we speculate that during prostate cancer metastasis the downregulation of miR-448 may contribute to the upregulation of SATB1, which may explain our observation that SATB1 expression was positively correlated with the bone metastasis of prostate cancer. Further studies are needed to test this possibility.

## Conclusions

SATB1 expression is positively correlated with the bone metastasis and the Gleason score of prostatic carcinoma. SATB1 overexpression promotes prostate cancer cell proliferation and invasion while SATB1 knockdown inhibits prostate cancer cell proliferation and invasion. These findings provide novel insight into the oncogenic role of SATB1 in prostate cancer, suggesting that SATB1 is a promising biomarker and therapeutic target for prostate cancer.

## Competing interests

The authors declare that they have no competing interests.

## Authors’ contributions

Conceived and designed the experiments: LM, JZ. Performed the experiments: LM, CY, JW. Analyzed the data: WL, RW. Contributed reagents/materials/analysis tools: JC. Wrote the paper: LW. All authors read and approved the final manuscript.
